# Metrological Set-Up and Procedure for Calibration of Displacement Transducers

**DOI:** 10.3390/s26206393

**Published:** 2026-10-09

**Authors:** Luka Čas, Bojan Ačko, Rok Klobučar

**Affiliations:** National Laboratory for Length in RS, Faculty of Mechanical Engineering, University of Maribor, Smetanova ulica 17, 2000 Maribor, Slovenia; bojan.acko@um.si (B.A.); rok.klobucar@um.si (R.K.)

**Keywords:** calibration, dimensional metrology, displacement transducer, automation, measurement uncertainty

## Abstract

High-precision displacement transducers are widely used in dimensional metrology due to their sub-micron accuracy and broad applicability. The increasing use of such sensors has led to a growing demand for SI-traceable calibration, requiring high-accuracy systems beyond the capabilities of commercial solutions. This paper presents the development of a dedicated calibration system for displacement transducers with measuring ranges up to 150 mm and its experimental evaluation over a 60 mm measurement range. The system is based on commercially available components and employs a laser interferometer as the reference standard, enabling an absolute measurement principle with direct traceability to the primary length standard. The calibration set-up is fully automated and designed to minimize dominant sources of systematic errors, particularly geometric misalignments including transducer alignment, interferometer beam alignment and Abbe error. A structured calibration procedure and a comprehensive measurement uncertainty model are presented to ensure repeatable and traceable calibration. Experimental evaluation was performed using a high-resolution displacement transducer with 60 mm measurement range in both directions of motion. The results demonstrate the repeatability and performance of the developed system over the experimentally investigated 60 mm measurement range and are consistent with the proposed uncertainty model. Based on this model, the expanded measurement uncertainty of the proposed calibration system is estimated to be 130 nm (*k* = 2) for measurement ranges of up to 150 mm.

## 1. Introduction

In modern industrial and research environments, high-precision displacement sensors, also known as transducers, have become indispensable tools in precise dimensional metrology [1]. These sensors, primarily inductive, capacitive or incremental, are widely used due to their ability to measure small displacements with sub-micrometer accuracy. They can operate either as stand-alone measurement instruments or as integrated components of more complex positioning and measurement systems [2,3]. Typical applications include the calibration of end standards (e.g., gauge blocks), precision positioning, surface roughness measurements and roundness measurements [4,5,6].

The increasing use of such sensors has created a growing need for high-accuracy calibration methods, traceable to the SI unit of length. As the national holder of the length standard, the Laboratory for Production Measurement at the University of Maribor (MIRS/UM-FS/LTM) is responsible for ensuring traceability of length measurements in Slovenia. In recent years, increasing demand has been observed from Slovenian industry and accredited calibration laboratories, as well as from neighbouring regions, for reliable calibration of high-precision displacement transducers with measuring ranges up to 150 mm. However, commercially available systems lack the required accuracy for these calibration tasks. Consequently, many leading metrology institutes have addressed this challenge by developing dedicated high-precision calibration systems.

A survey of the Key Comparison Database (KCDB) [7], maintained by the Bureau International des Poids et Mesures (BIPM) [8], shows that only ten national metrology institutes (NMIs) currently hold internationally recognized Calibration and Measurement Capabilities (CMCs) for displacement transducer calibrations over the measurement range relevant to this work. This highlights the relatively small number of institutes worldwide capable of performing such calibrations. A review of the literature indicates that these institutes rarely publish their procedures, reflecting the proprietary nature of high-precision metrology and the emphasis on protecting commercial or competitive advantage. One notable exception is the recent conducted EURAMET supplementary comparison EURAMET.L-S2.1.n01 [9], involving leading European national metrology institutes to demonstrate and compare their high-accuracy displacement transducer calibration capabilities. However, the report primarily presents the comparison results and does not provide detailed descriptions of the calibration systems, measurement procedures, or uncertainty evaluation methodologies implemented by the participating laboratories.

Beyond the activities of NMIs, several calibration solutions have nevertheless been reported in the literature. Early approaches relied primarily on universal mechanical systems, such as single-axis measuring machines or coordinate measuring machines (CMMs), enabling one-dimensional measurements. While these systems can provide accurate displacement generation, their measurement uncertainty is typically limited by the mechanical characteristics of the machine and by geometric alignment errors. Growing demands for reduced measurement uncertainty have driven rapid development in this field, with most laboratories following one of two main approaches: building dedicated calibration systems or upgrading existing 1D universal machines and CMMs with integrated laser interferometers [10,11,12].

A typical dedicated calibration system, as presented in [1], consists of a movable calibration platform to which the sensor under test is attached. The platform is driven over the desired measurement range, and the sensor displacement is recorded simultaneously with readings from a reference sensor. The reference sensor is typically placed on the opposite side of the platform relative to the sensor under test, but it can also measure the same surface from the same side. Configurations may be oriented either vertically or horizontally.

In [13,14,15], the authors present prototype systems with horizontal and vertical configurations using a He-Ne laser interferometer as the reference standard. A notable feature is the active compensation of angular deviations of the positioning guideways using photodetectors and a three-axis PZT tilt system to maintain perpendicular alignment of the measuring mirror with the laser beam.

Other systems described in [3,16,17] are based on micrometric linear stages mounted on invar base plates, incorporating high-flatness mirrors and piezoelectric actuators for angular alignment. Semi-automated calibration systems have also been reported [18], where displacement is generated by motorized linear guideways and the measurement is performed using linear encoders under LabVIEW control.

The second main approach involves upgrading existing universal measuring machines with laser interferometers [19,20,21]. These systems typically focus on minimizing geometric errors such as Abbe and cosine errors, while also addressing thermal and mechanical influences through appropriate mechanical design and mounting strategies.

Across the reviewed studies [3,13,14,15,16,17,18,19,20,21], most calibration systems share similar core components: a displacement generation mechanism, a mounting structure for the sensor under test, and a reference measurement system. Laser interferometers based on stabilized He–Ne lasers are widely used as reference instruments [22]. The literature further indicates that dominant sources of measurement uncertainty include geometric misalignments, particularly Abbe and cosine errors, as well as structural stiffness, guideway alignment and flatness of the contact surface used for sensor calibration.

Environmental conditions represent another important source of measurement uncertainty in dimensional measurements. Temperature, pressure and humidity variations influence both the mechanical structure of the system and the refractive index of air. To mitigate these effects, many laboratories perform measurements in temperature-controlled chambers. Highly stable environments with temperature stability better than ±0.01 K have been reported at NMIs such as NIST [23], while similar facilities with stability below ±0.05 K are described in [24]. However, such solutions are often associated with high implementation and operating costs.

Despite the reported developments in high-accuracy calibration of one-dimensional displacement transducers, important gaps remain in the published literature [3,9,13,14,15,16,17,18,19,20,21]. In particular, detailed descriptions of calibration system implementation and measurement procedures are limited, while comprehensive measurement uncertainty evaluations are often missing or only partially addressed. Furthermore, the quantitative contribution of alignment procedures and individual error sources to the overall measurement uncertainty is not consistently reported. Although some automated or semi-automated measurement solutions have been demonstrated [9,13,14,18], many reported calibration approaches still rely on manual operations, and fully automated calibration workflows remain less commonly described. These aspects can limit the reproducibility and independent assessment of calibration procedures and highlight the need for more comprehensive, well-characterized and automated approaches.

This paper presents the development of a dedicated calibration system for high-precision contact-type, one-dimensional displacement transducers, including dial gauges, addressing the identified limitations. The system is based on commercially available components and employs a laser interferometer as the reference measurement standard [10,11]. The approach is based on an absolute measurement principle, ensuring direct traceability to the primary length standard. The use of laser interferometry as a reference measurement technique is well established and is therefore not claimed as a novel aspect of this work. Rather, the novelty lies in the development and systematic metrological characterization of an integrated, traceable calibration system and procedure. The main contributions include the following:

- Systematic metrological characterization of the calibration system, including the evaluation and control of the main geometric and environmental error sources;
- A vision-based approach for quantitative alignment of the transducer measurement axis with the direction of motion defined by the motorized translation stage;
- An automated bidirectional calibration procedure with user-defined measurement sequences, points, and repetitions;
- A comprehensive measurement uncertainty evaluation in accordance with the GUM.

The developed uncertainty model indicates that the proposed automated calibration system is capable of achieving an expanded measurement uncertainty of 130 nm (*k* = 2) over a displacement range of up to 150 mm. The developed system was experimentally evaluated over a 60 mm measurement range using a high-resolution displacement transducer in both directions of motion.

## 2. Materials and Methods

### 2.1. Calibration System Design

The developed calibration system comprises a mechanical structure, a reference measurement system, and environmental control, which are described in the following subsections.

#### 2.1.1. Mechanical Structure

Most high-precision displacement transducers, particularly those with larger measurement ranges, are intended for use in a vertical measurement position. Accordingly, the reference calibration set-up has been configured with a vertical mounting of the transducer. Performing the calibration in this orientation also eliminates the effect of the transducer shaft sag on the calibration results and the associated measurement uncertainty. The mechanical structure is further designed to provide multiple adjustment mechanisms for precise alignment and positioning. Most of the components used in the set-up were commercially available products supplied by Thorlabs, while some components were manufactured in-house.

**Figure 1:**
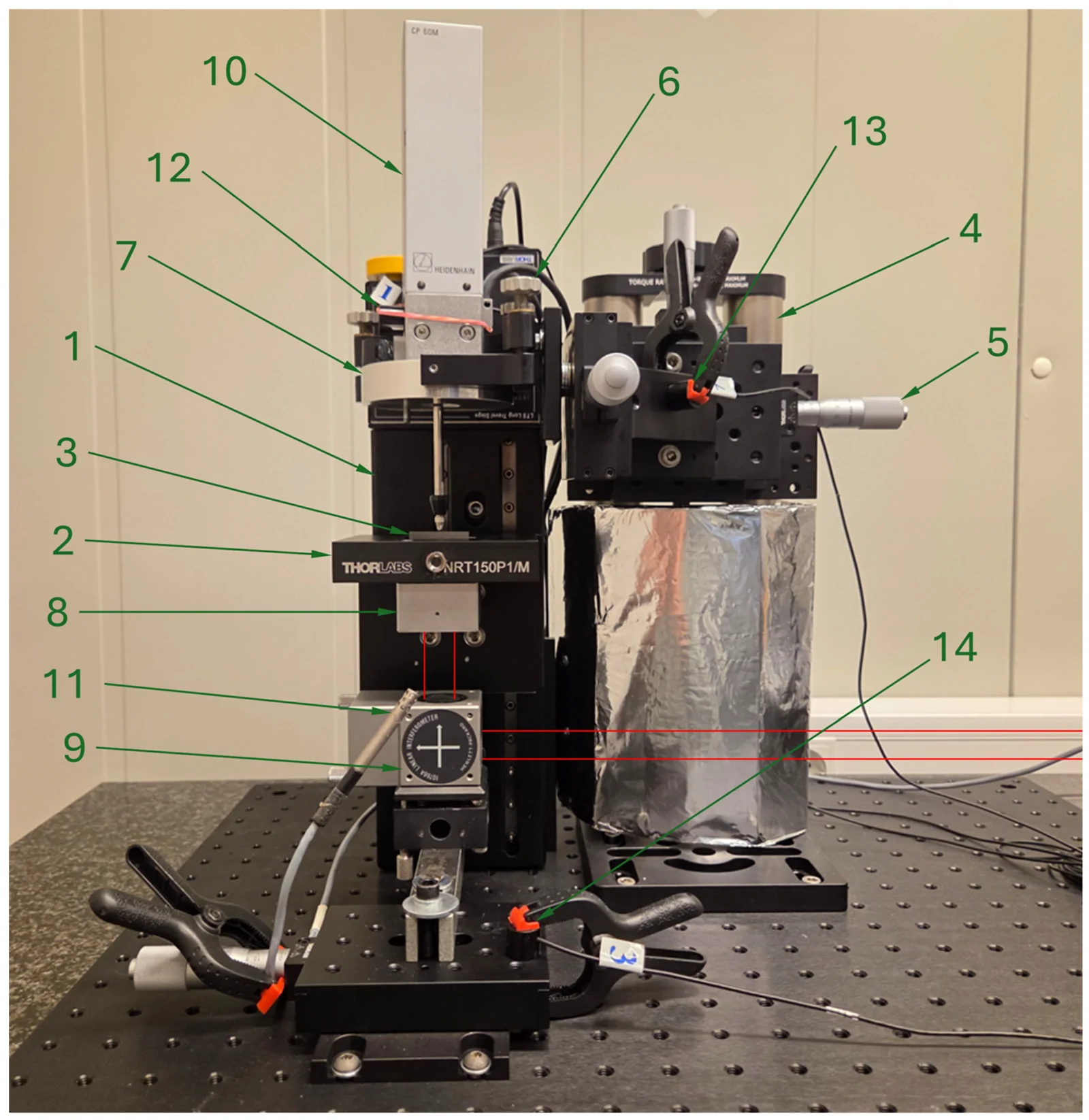
Experimental set-up of the vertical-axis calibration system: (1) motorized linear translation stage, (2) calibration platform, (3) steel gauge block, (4) vertical translation stage, (5) manual micropositioning stage, (6) 3-axis rotary adjustable mount, (7) cylindrical transducer holder, (8) movable retroreflector, (9) fixed interferometer optics, (10) transducer under test, (11) air temperature sensor, and (12–14) material temperature sensors.

The developed vertical-axis calibration system is shown in Figure 1. The core displacement-generating mechanism consists of a motorized linear translation stage (1), with 150 mm travel range, vertically mounted on an aluminium base plate, fixed to a granite surface plate for enhanced rigidity and vibration damping. The stage provides a minimum incremental motion of 100 nm and a bidirectional repeatability of less than 2 µm. It can be precisely controlled via computer, allowing automated and reproducible positioning. A calibration platform (2) is mounted on the translation stage, used to transfer motion to the test sensor. A steel gauge block (3) is placed between the platform and the transducer tip, ensuring a high-flatness contact surface for stable and reproducible displacement transfer.

Parallel to the main station, a vertical translation stage (4) is mounted, carrying a micropositioning manual stage (5) that allows micrometric translational movement along three axes. Attached to this stage is a 3-axis rotary adjustable mount (6), originally designed for optical components, but in this case repurposed to hold the displacement transducer under test. This mount enables fine rotational alignment in three axes (±5°), which is essential for accurate transducer positioning. A cylindrical holder (7) with a through-hole is attached to the mount, providing a secure fixation for the test sensor.

Based on a survey of commercially available transducers [6,25], two main types of mounting interfaces were identified: the first type features a cylindrical shaft (Ø 8 mm) suitable for set-screw fixation, while the second type requires screw-based clamping. Accordingly, two dedicated holders were designed to accommodate both interface types, ensuring reliable mounting of the sensors during calibration.

#### 2.1.2. Reference System

The reference measurement is provided by a laser interferometer, chosen for its high resolution and traceability. In our set-up, an HP 5528A laser interferometer (Hewlett-Packard, Spring, TX, USA) [26,27] available in the lab is employed. The interferometer’s laser frequency is calibrated against a primary length standard in the laboratory [28,29], while the integrated environmental sensor is calibrated at the national reference laboratories for temperature and pressure.

A laser interferometer is positioned coaxially on the opposite side of the calibration platform relative to the sensor under test, enabling direct comparison of measured displacements along the same measurement axis with minimized Abbe error. A movable retroreflector (8) is mounted on the platform using screws. The fixed part of the interferometric optics (9) is mounted to the base plate via a dedicated support, which allows manual adjustment in one translational direction as well as rotational alignment. All interferometric optics used in the setup belong to the HP 5528A laser interferometer system. The laser head is not shown in Figure 1 because it is positioned sufficiently far from the calibration system to prevent heat generated by the laser head from affecting the measurement system. The laser beam path is indicated by red lines.

#### 2.1.3. Environmental Control

The entire set-up operates within a controlled laboratory chamber featuring active temperature regulation (±0.1 K stability) and continuous humidity monitoring. Temperature sensors are used to monitor the air inside the chamber as well as the mechanical structure and the sensor under test. For precise monitoring of both air and material temperatures, a JENAer Meßtechnik TEMP 14 system [30] is employed, providing a measurement uncertainty of *U* = 15 mK; *k* = 2 for material sensors and *U* = 50 mK; *k* = 2 for the air sensor. Traceability of these sensors is ensured by the National laboratory for temperature in Slovenia (MIRS/ULFE/LMK). Atmospheric pressure is monitored using the integrated pressure sensor of the HP 5528A laser interferometer system, with a measurement uncertainty of *U* = 0.01 kPa; *k* = 2.

### 2.2. Calibration Procedure

The calibration procedure consists of three main stages. First, the transducer and the calibration system are prepared and thermally stabilized to ensure stable measurement conditions. Second, the dominant geometric error sources, including transducer alignment, laser beam alignment, and the Abbe error, are minimized. Finally, the automated calibration measurements are performed by generating a defined displacement while simultaneously recording the indication of the transducer under test and the corresponding reference displacement measured by the traceable laser interferometer. The individual steps of the procedure are described in detail in the following sections.

#### 2.2.1. Preparation Before Measurement

Upon receipt, the transducer under test is visually inspected for any mechanical damage, such as scratches or corrosion, that could affect the calibration results. Each sensor is identified and documented prior to further processing. All relevant contact surfaces, such as the measuring surface of the transducer and the contact surface on the calibration platform (gauge block), are carefully cleaned using petroleum ether, while optical components of the interferometer are cleaned using appropriate lint-free materials.

After cleaning, the transducer is mounted into the calibration system, and a preliminary (coarse) alignment is performed. This step ensures that the sensor axis is approximately aligned with the direction of motion and that the contact conditions between the sensor and the calibration platform are properly established. Thermal stabilization of the sensor under test is then performed in a temperature-controlled environmental chamber.

**Figure 2:**
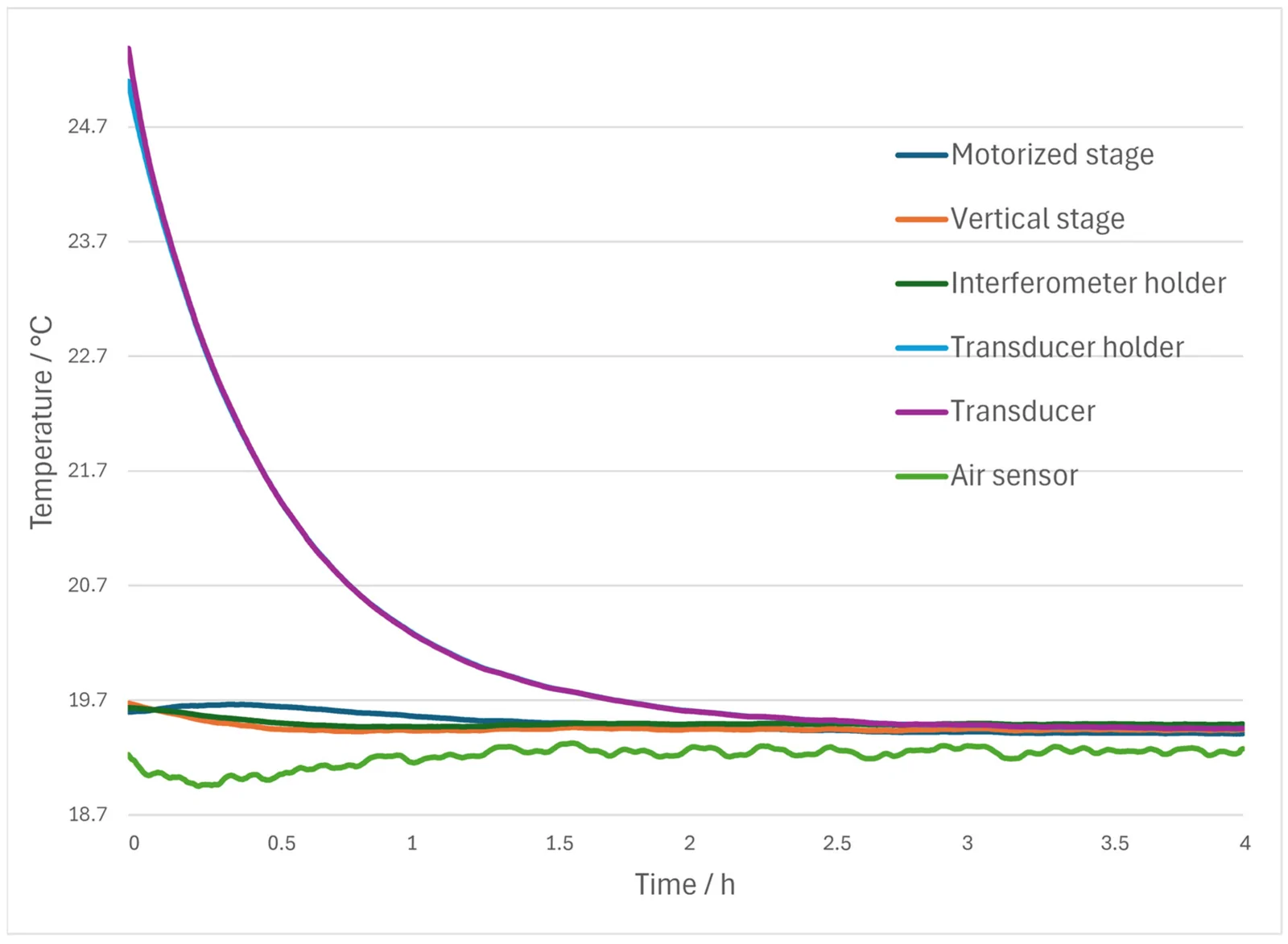
Temperature stabilization of the transducer and its holder compared with the other calibration system components, from an initial temperature of approximately 25 °C, showing the convergence of material and air temperatures towards thermal equilibrium.

The stabilization behaviour was experimentally evaluated to determine the required temperature stabilization time of the transducer. For this purpose, five material temperature sensors and one air temperature sensor were used to monitor the transducer, the transducer holder, the mechanical structure of the calibration system and the surrounding air. Specifically, the material temperature sensors were attached to the transducer, the transducer holder, the vertical translation stage, the interferometer holder, and the motorized translation stage. For the stabilization test, the transducer and its holder initially exhibited a temperature of approximately 25 °C. This initial temperature was selected as a conservative condition to represent a possible initial thermal state of the transducer after receipt and handling during mounting. Since the transducer and its holder are directly handled during the preparation and mounting procedure, they may initially have a higher temperature than the remaining components of the calibration system. The selected initial condition was therefore intentionally conservative to ensure that the determined stabilization time provides sufficient margin for achieving stable thermal conditions. The evolution of temperatures during stabilization is shown in Figure 2.

The system was considered thermally stabilized when the temperature of the transducer and the monitored mechanical components became stable. Based on this criterion, the transducer and the system reached thermal equilibrium after approximately 3 h, indicating stable thermal conditions.

Following thermal stabilization, a fine alignment of the system is performed to minimize geometric measurement errors. This step includes precise adjustment of the sensor orientation and the interferometer measurement axis.

As the alignment procedure involves manual adjustments, a short additional stabilization period of approximately 1 h is applied to minimize the influence of thermal disturbances introduced during handling. After this final stabilization step, the system is considered ready for calibration measurement.

#### 2.2.2. Transducer Alignment

**Figure 3:**
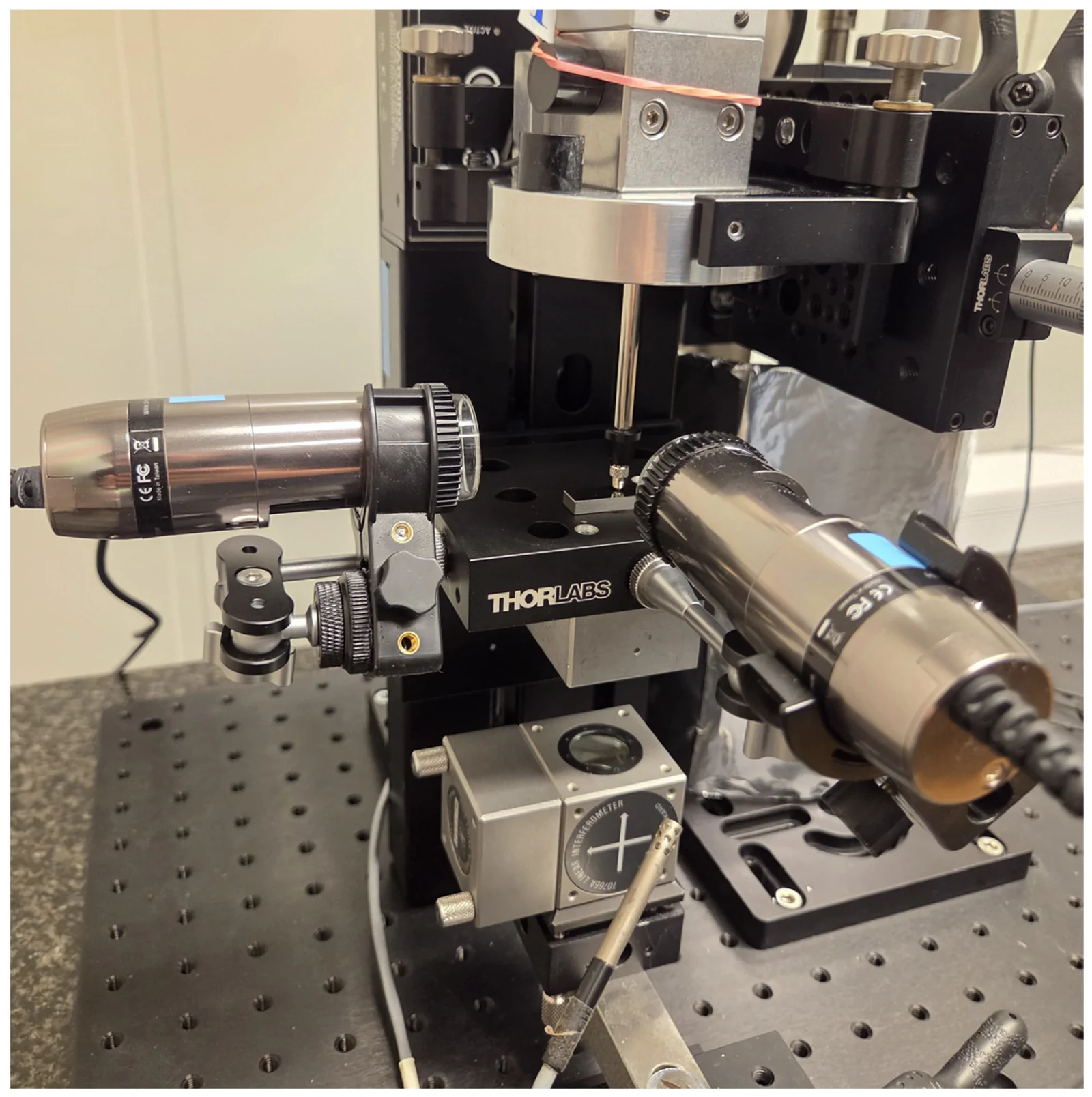
Vision-based system for transducer alignment with two orthogonally arranged cameras.

Minimization of cosine error requires precise alignment of the transducer axis with the direction of motion defined by the motorized translation stage. For this purpose, a non-contact vision-based alignment system is employed, using two digital cameras mounted directly on the translation stage, as shown in Figure 3. Recent work has also demonstrated camera-based optical techniques for high-resolution displacement measurement [31]. The cameras are arranged orthogonally to each other and perpendicular to the direction of motion, enabling independent observation of the sensor alignment in two perpendicular planes. This configuration ensures that any angular deviations of the guideways are inherently captured within the measurement frame.

The alignment is performed by detecting the edge of the transducer shaft using grayscale image processing with subpixel edge detection. By acquiring images at different positions along the measurement range, the lateral displacement of the detected edge is evaluated, allowing estimation of the angular deviation of the sensor relative to the direction of motion. The required correction is then applied through manual adjustment of the 3-axis rotary adjustable mount. The procedure is iteratively repeated until the residual angular deviation is minimized.

A vision-based alignment approach is currently under development and shows promising potential for improving alignment sensitivity beyond conventional methods. While a full validation is outside the scope of this paper, preliminary results indicate a significant reduction in alignment uncertainty, with an expected alignment accuracy on the order of 2 µm over a 30 mm length.

For comparison, conventional alignment using a dial gauge [32] enables achieving a residual lateral deviation of approximately 3 µm over a 10 mm displacement.

#### 2.2.3. Laser Beam Alignment

The alignment of the interferometer is performed in two steps. First, the interferometric optical components are aligned with the measurement axis. This is achieved using a dial gauge fixed on the motorized vertical stage, allowing adjustment in both lateral directions (x and y). The alignment is iteratively refined until the variation in the dial gauge readings along the optical components does not exceed 2 µm.

In the second step, the laser beam is aligned with the direction of motion of the linear guides. For this purpose, a 2D position-sensitive optical detector is mounted at the location of the moving retroreflector. The detector measures the lateral position of the laser beam and provides real-time feedback during stage movement. By adjusting the position and orientation of the laser head, the beam is aligned until no measurable lateral displacement is observed over the full travel range.

#### 2.2.4. Abbe Error

**Figure 4:**
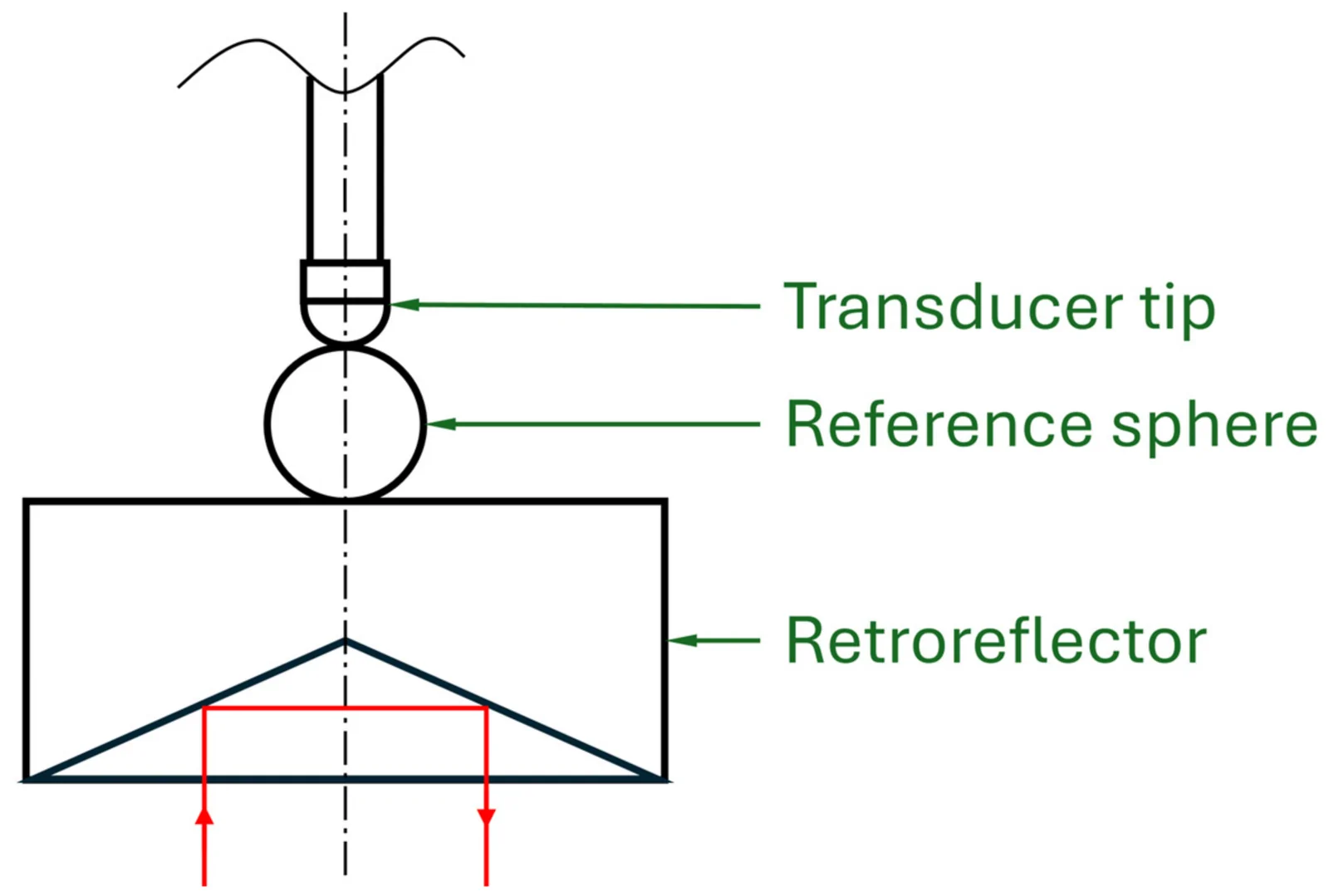
Schematic of Abbe error minimization by near-coaxial alignment of the transducer and interferometer measurement axes. The alignment is performed using a reference sphere to locate the optical axis by maximizing the transducer signal.

The Abbe error is minimized by ensuring near-coaxial alignment between the measurement axes of the laser beam and the transducer, as shown in Figure 4. The remaining contribution is evaluated based on the angular behaviour of the translation stage.

This behaviour was experimentally characterized by measuring pitch and yaw of the translation stage over the full measurement range using interferometric angular optics.

To ensure coaxial alignment between the transducer axis and the interferometer measurement axis, a dedicated reference artefact was used to define the centre of the interferometer optics. A precision sphere was mounted at the centre of the retroreflector, and its position was measured using a ZEISS UMC 850 coordinate measuring machine (CMM) (Carl Zeiss, Jena, Germany). The deviation of the sphere centre from the optical axis was determined to be within 30 µm in both lateral directions.

During alignment, the spherical tip of the transducer is brought into contact with the reference sphere and manually adjusted in the lateral directions (x and y). The transducer output signal is monitored and the position corresponding to the maximum reading is identified along both axes. This position corresponds to the centre of the sphere, enabling alignment of the transducer axis with the interferometer axis. Using this method, a lateral alignment accuracy better than 0.1 mm is achieved.

#### 2.2.5. Measurement Procedure

**Figure 5:**
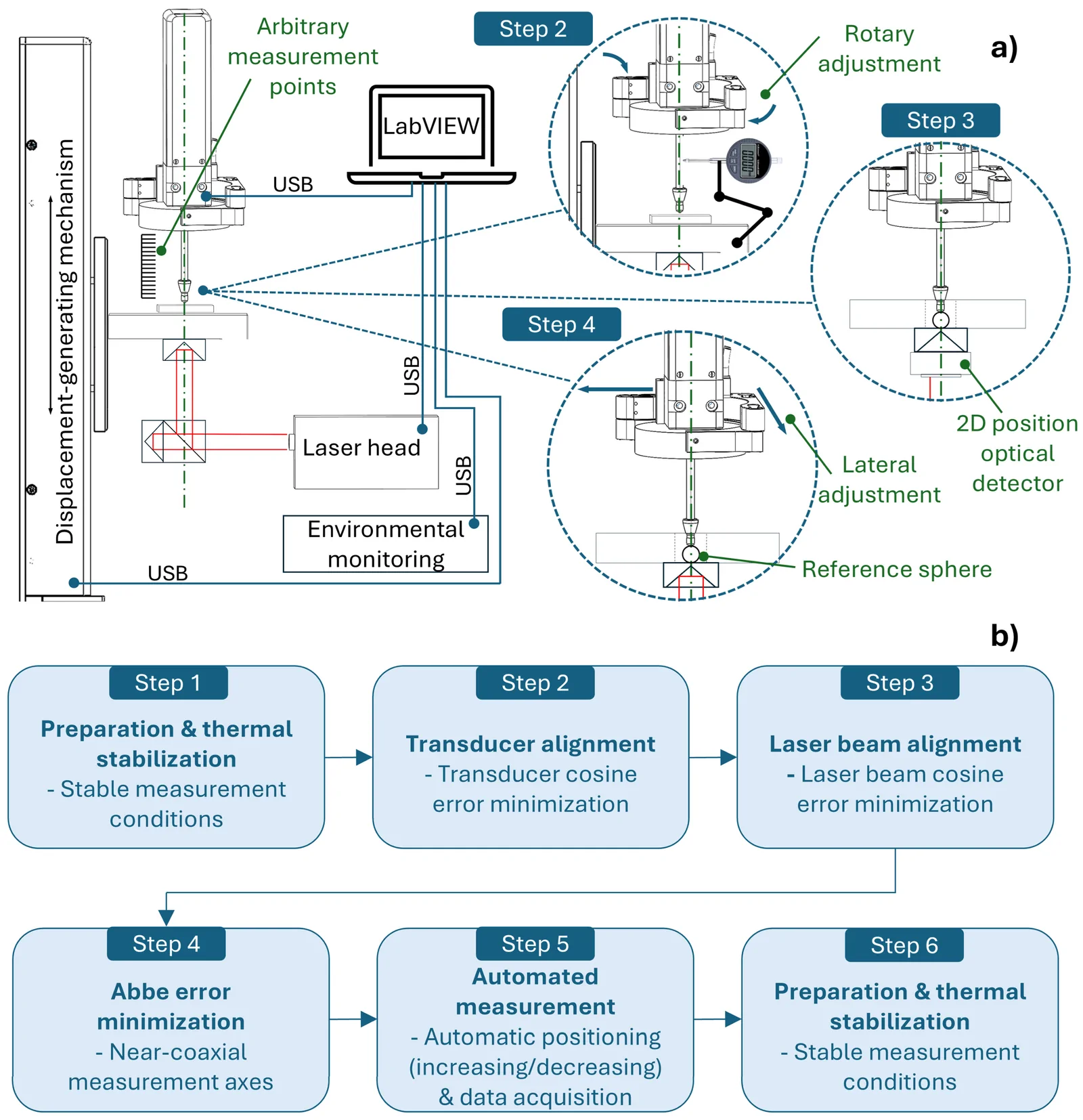
Schematic representation of the calibration principle and procedure: (**a**) measurement configuration and main alignment procedures; (**b**) calibration procedure flow chart.

The overall calibration principle and measurement procedure are illustrated in Figure 5. After completion of the alignment procedures and thermal stabilization, the calibration measurement is performed in an automated sequence.

The calibration system allows the selection of arbitrary measurement points and a desired number of repetitions, based on client requirements. The measurement sequence is fully controlled by a dedicated LabVIEW 2023 Q1 (32-bit) application, which automatically positions the translation stage at each predefined measurement point, acquires the transducer and laser interferometer signals, and stores the recorded data for further evaluation. The minimum step between measurement points can be set as low as 1 µm. The calibration procedure is performed using a low displacement speed of 1 mm/s and a maximum acceleration of 1 mm/s^2^ to minimize possible dynamic effects. Additional tests at higher motion speeds confirmed that no significant influence on the calibration results was observed.

The linear translation stage is used solely to generate the required displacement and is therefore not considered a measurement instrument. The actual displacement is determined directly using the traceable laser interferometer. Calibration is performed at discrete measurement positions. At each position, the translation stage is brought to the target position and allowed to stabilize before data acquisition. At each measurement position, the indication of the transducer under test and the corresponding reference displacement measured by the traceable laser interferometer are acquired simultaneously. The difference between these values forms the basis for the calibration result, which is subsequently evaluated according to the mathematical model presented in Section 3.1.

The LabVIEW application also continuously monitors the transducer and laser interferometer signals together with the environmental parameters, including air temperature, humidity, pressure, and the temperatures of the mechanical structure and the transducer under test. Linear thermal expansion of the transducer material can also be entered for automatic compensation.

The calibration procedure is performed in both the increasing and decreasing directions of motion. Calibration begins by defining the zero point. The vertical translation stage is moved to the position where the transducer and the gauge block come into contact. Both the transducer and the LI are then reset to establish the reference zero. Before starting the measurement sequence, the stage is retracted by 0.5 mm, ensuring that the transducer approaches the measurement point in the increasing direction. The system then performs the complete sequence of predefined measurement points. After completion of each sequence, the translation stage moves 0.5 mm beyond the zero point and then returns to the zero point. This ensures that the zero point is always approached from the increasing direction, preventing hysteresis effects. Both the transducer and the LI are then reset to eliminate zero-point drift before the next repetition. This procedure is repeated for the selected number of repetitions. For the decreasing direction, the same procedure is followed, with the measurement sequence performed in the decreasing direction. Positioning can be performed either relative to the translation stage, the transducer under test, or the reference LI.

For each measurement point, the recorded values from all repetitions are averaged, and the results from all sensors are stored in an organized xlsx file from which the calibration certificate is created. The fully automated measurement sequence ensures high repeatability and minimizes the influence of random effects.

## 3. Results

The Results Section presents the evaluation of the measurement uncertainty according to the GUM, followed by the experimental evaluation of the developed calibration system.

### 3.1. Measurement Uncertainty

The calibration result is defined as the deviation between the displacement measured by the LI and the indication of the transducer under calibration, corrected for relevant systematic effects. The mathematical model is expressed by Equation (1):

(1) $$e=\left(L_{LI}+e_{cos}+e_{dp}\right)-L_{T}\cdot \left(1+\alpha_{T}\cdot \theta_{T}\right)-e_{cosT}-e_{Abbe}-e_{re}$$

where
•  edeviation between LI and transducer (calibration result) at 20 °C;•  $L_{T}$reading of the transducer under test;•  $\alpha_{T}$the linear thermal expansion coefficient of the transducer;•  $\theta_{T}$transducer temperature deviation from 20 °C;•  $L_{LI}$corrected displacement indicated by LI;•  $e_{cos}$cosine error due to laser beam adjustment, assumed to be 0 based on alignment procedure;•  $e_{dp}$dead path error, assumed to be 0;•  $e_{cosT}$cosine error due to transducer misalignment, assumed to be 0 based on alignment procedure;•  $e_{Abbe}$Abbe error due to angular deviations (pitch and yaw) of the translation stage;•  $e_{re}$random errors/repeatability.

The combined standard uncertainty of the calibration result is evaluated according to the Guide to the Expression of Uncertainty in Measurement (GUM) [33] using the law of propagation of uncertainty, as given in Equation (2):

(2) $$u_{c}^{2}(e)=\sum c_{i}^{2}\;u^{2}(x_{i})$$

where $c_{i}$ is the sensitivity coefficients obtained as partial derivatives of the measurement model and $u(x_{i})$ represents the corresponding standard uncertainties of the input values. These uncertainties are determined based on the characteristics of the reference system, the calibration procedure and the assumed measurement conditions. In the following sections, the individual uncertainty contributions are described in detail.

#### 3.1.1. Uncertainty of the Transducer Reading *u*(*L*_T_)

The uncertainty associated with the reading of the transducer arises from its finite resolution. For a digital transducer with a resolution of 10 nm, the maximum quantization error is ±5 nm. Assuming a rectangular distribution of this error, the corresponding standard uncertainty is 3 nm.

#### 3.1.2. Uncertainty of the Transducer Temperature Expansion Coefficient *u*(*α*_T_)

The effect of thermal expansion of the transducer is corrected using the coefficient of thermal expansion $\alpha_{T}$. The temperature of the transducer is monitored during calibration, while the corresponding expansion coefficient is either taken from manufacturer data or estimated from reference material properties. The uncertainty associated with $\alpha_{T}$ is estimated by assuming a possible deviation of $\pm 1\cdot 10^{-6}\;K^{-1}$ from the nominal value. Assuming a rectangular distribution, the corresponding standard uncertainty is $0.58\cdot 10^{-6}\;K^{-1}$.

#### 3.1.3. Uncertainty of the Transducer Temperature Deviation *u*(*θ*_T_)

The temperature of the transducer holder is measured during calibration using a contact temperature sensor. This is necessary for transducer types where direct temperature sensor attachment is not possible due to geometry constraints or limited available surface for sensor mounting. It is assumed that the transducer is in thermal equilibrium with the holder after thermal stabilization. The validity of this assumption was experimentally evaluated during the thermal stabilization procedure described in Section 2.2.1, where the temperatures of both the transducer and the transducer holder were monitored simultaneously. The experiment showed that thermal equilibrium was reached after approximately 3 h, followed by an additional stabilization period of approximately 1 h after the alignment procedure.

The uncertainty of the transducer temperature is therefore evaluated from three contributions: the calibration uncertainty of the temperature sensor, observed temperature variation during the measurement, and the possible temperature difference between the holder and the transducer. The calibration uncertainty of the temperature sensor is U=0.015 K;k=2, corresponding to a standard uncertainty of 0.0075 K.

The observed temperature variations during the measurement process remained within ±0.01 K based on experimental monitoring, while the maximum measured temperature difference between the holder and the transducer was ±0.03 K. Assuming rectangular distributions for the latter two contributions, the combined standard uncertainty is 0.02 K.

#### 3.1.4. Uncertainty of the Laser Interferometer Indication *u*(*L*_LI_)

The uncertainty of the laser interferometer (LI) indication is evaluated based on its calibration history, considering calibration deviations, the uncertainty of previous calibrations, and long-term drift. The resulting standard uncertainty is expressed as a combination of a constant and a length-dependent term: $u\left(L_{LI}\right)=10\;nm+0.2\cdot 10^{-6}\cdot L$.

The effective laser wavelength is corrected for variations in air temperature, pressure, and humidity by applying the Edlén equation. A detailed analysis of the influence of environmental conditions on the laser interferometer uncertainty under the conditions of the environmental enclosure used in this work has been presented previously [32,34].

#### 3.1.5. Uncertainty Due to Dead Path Error of the LI *u*(*e*_dp_)

The dead path error arises from changes in air conditions between the laser interferometer initialization and the measurement position. The optical path difference between the interferometer and the moving retroreflector is less than 0.5 mm, effectively minimizing this effect. Experimental evaluation of the system showed a corresponding standard uncertainty of 9 nm.

#### 3.1.6. Uncertainty Due to LI Cosine Error *u*(*e*_cos_)

The cosine error of the laser interferometer is minimized through careful alignment of the laser beam with the direction of motion, as described in Section 2.2.3. Based on experimental evaluation, the maximum residual cosine error is estimated to be 50 nm over the full measurement range. Assuming a rectangular distribution, the corresponding standard uncertainty is $3\cdot 10^{-7}\cdot L$.

#### 3.1.7. Uncertainty Due to Transducer Cosine Error *u*(*e*_cosT_)

The residual cosine error of the transducer is primarily determined by the achieved alignment. Using a dial indicator, a lateral deviation of approximately 3 µm over a 10 mm shaft can be attained, corresponding to a cosine error of $e_{cosT1}=4.5\cdot 10^{-8}\cdot L$. Considering two perpendicular axes (x and y) and rounding up, the total cosine error is $e_{cosT}=1\cdot 10^{-7}\cdot L$. Standard uncertainty, assuming a rectangular distribution, is $6\cdot 10^{-8}\cdot L$.

A vision-based alignment system using cameras is currently implemented. While this system is expected to provide improved alignment, its performance has not yet been fully validated. Therefore, the uncertainty assessment conservatively relies on the alignment achievable with the dial indicator.

#### 3.1.8. Uncertainty Due to Abbe Error *u*(*e*_Abbe_)

The Abbe error arises from angular deviations of the translation stage, producing a lateral displacement proportional to the distance between the transducer and the interferometer axis. As described in Section 2.2.4, the geometric deviations of the linear stage were evaluated using the laser interferometer and angular optics. The maximum angular deviation measured along the full travel range was 40 µm/m in pitch and 30 µm/m in yaw. Assuming a rectangular distribution and an Abbe offset of 0.1 mm, the corresponding standard uncertainty is 3 nm for pitch and 2 nm for yaw, resulting in a combined standard uncertainty of 4 nm.

#### 3.1.9. Uncertainty Due to Random Errors *u*(*e*_re_)

Random errors arise from small variations in the measurement system and environmental conditions. These effects are reduced by performing repeated measurements at each calibration point. For each position, the measurement is repeated 30 times. The experimentally determined standard deviation in the worst-case scenario was 12 nm. Considering 30 repeated measurements at each calibration point, the corresponding standard uncertainty is 2 nm.

#### 3.1.10. Expanded Uncertainty

The expanded uncertainty is evaluated using a coverage factor k=2, corresponding to a confidence level of approximately 95%. This choice follows standard practice in dimensional metrology and is justified by the GUM, as the uncertainty budget is heavily dominated by Type B components, resulting in large effective degrees of freedom. The best achievable expanded uncertainty of the calibration system is expressed in Equation (3). The complete uncertainty budget is summarized in Table 1.

(3) $$U=\sqrt{\left(29\;\mathrm{nm}{)}^{2}+(8.4\cdot 10^{-7}\cdot L{)}^{2}\right.}$$

### 3.2. Experimental Evaluation

To evaluate the performance of the developed calibration system and assess the consistency of the experimental results with the estimated measurement uncertainty, measurements were performed using a Heidenhain Certo CP 60M displacement transducer with a measuring range of 60 mm and a resolution of 10 nm. Although its measuring range is limited to 60 mm, it represents the highest-accuracy transfer standard available for experimental evaluation of the proposed calibration system. The transducer is based on a Zerodur measurement scale; as the thermal expansion coefficient is not specified by the manufacturer, its influence was assumed negligible due to the inherently low thermal expansion of Zerodur. The transducer operates without an internal return spring, with the measurement force generated solely by gravity. Its effective measurement force was experimentally evaluated using a dial tension gauge and remained stable over the complete measurement range, with values of (0.5 ± 0.02) N during inward motion and (0.4 ± 0.02) N during outward motion.

Measurements were performed at 29 discrete positions distributed along the 60 mm range. The measurements were carried out in both increasing and decreasing directions of motion to evaluate hysteresis effects. At each measurement position, 30 measurement sequences were performed. For each direction of motion, the calibration error represents the average result obtained from these 30 sequences.

**Figure 6:**
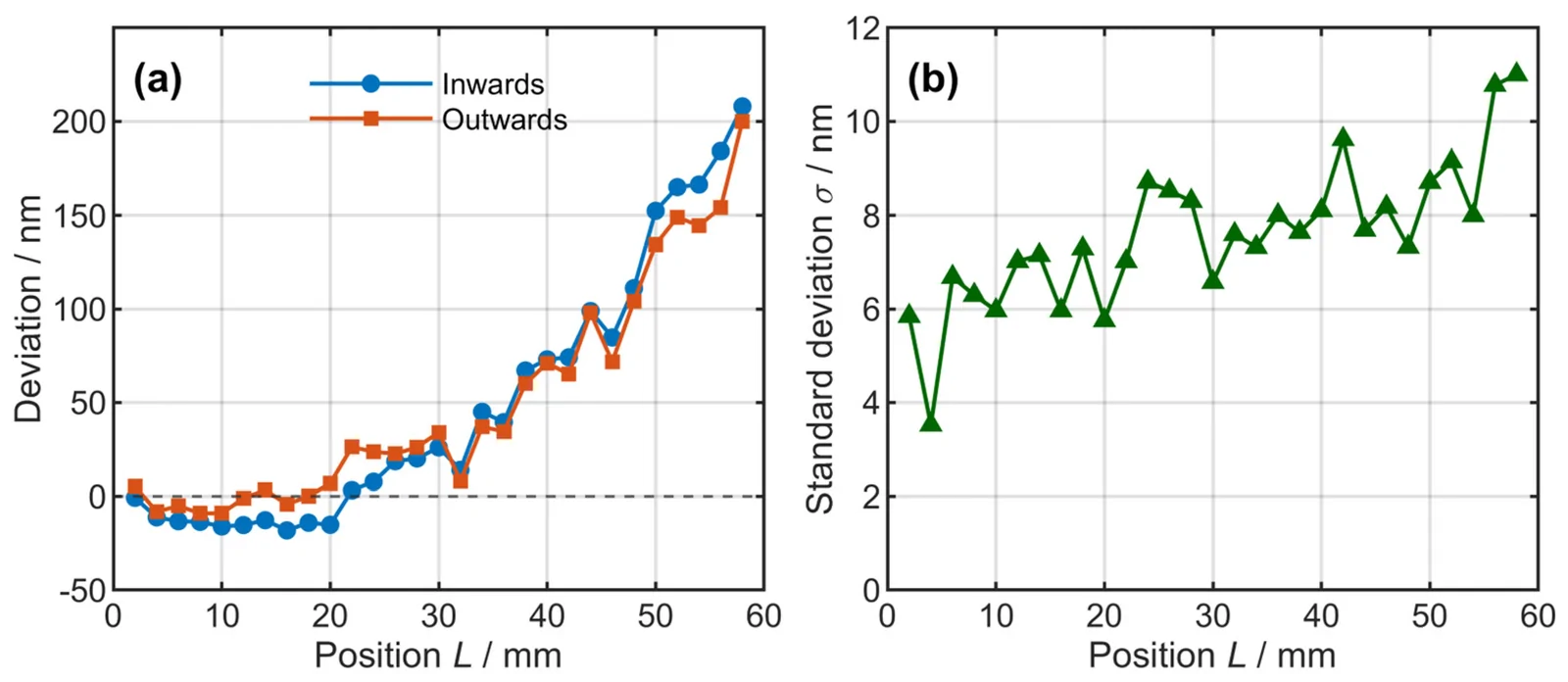
Experimental evaluation of the displacement transducer under test over the 60 mm measurement range: (**a**) averaged calibration error (*n* = 30) for both directions of motion; (**b**) standard deviation of the averaged bidirectional measurement results (*n* = 30).

The measurement results are shown in Figure 6a, where the resulting calibration error of transducer under test is presented for both directions of motion. Good agreement is observed between increasing and decreasing runs, with a small hysteresis effect, where the maximum reverse error does not exceed 30 nm.

Figure 6b presents the standard deviation of the average bidirectional measurement results obtained from the same 30 measurement sequences at each measurement position. For each measurement sequence, the results obtained in the increasing and decreasing directions were averaged to obtain one bidirectional measurement result. The standard deviation was then calculated from the 30 bidirectional results at each position. This value serves as an indicator of the repeatability of the developed calibration system. A slight increase in the measurement spread with displacement can be observed, although the trend is not strictly linear. The maximum standard deviation does not exceed 12 nm over the entire measurement range.

The experimental results demonstrate the repeatability of the developed calibration system and are consistent with the estimated measurement uncertainty.

## 4. Discussion

A dedicated calibration system for displacement transducers with a measuring range up to 150 mm has been developed. The system performance has been experimentally evaluated along a limited measurement range of 60 mm using a Heidenhain Certo CP 60M displacement transducer with a resolution of 10 nm. The main objective of this work has been achieved through the development of a cost-effective and fully automated system, with GUM-based evaluation indicating an expanded measurement uncertainty of $U={\left((29\;\mathrm{nm}{)}^{2}+(8.4\cdot 10^{-7}\cdot L{)}^{2}\right)}^{1/2}$, supporting its capability to achieve performance comparable to leading national metrology institutes. The experimental evaluation over 60 mm demonstrates the performance of the developed system within the experimentally accessible range, while the uncertainty evaluation supports its potential for high-accuracy calibration over the full 150 mm range.

The system employs a laser interferometer as the reference standard and is based on commercially available components, ensuring direct traceability through an absolute measurement approach. The use of laser interferometry as a reference measurement technique is well established and provides a highly accurate and traceable measurement basis; therefore, it is not considered a novel aspect of the present work. Rather, the contribution lies in the integrated development and systematic metrological characterization of the calibration system and procedure. The design addresses the dominant systematic error sources, including geometric misalignments such as transducer alignment, interferometer beam alignment, and Abbe error, and incorporates a quantitative vision-based alignment procedure for accurate alignment of the transducer measurement axis with the direction of motion. The calibration procedure is fully automated and enables repeated bidirectional measurements at multiple discrete positions across the measurement range. In addition, a comprehensive GUM-based measurement uncertainty model was developed, with the major uncertainty contributions identified and quantified.

Experimental results using a high-resolution displacement transducer demonstrated good repeatability and consistent calibration performance over the investigated 60 mm range. A comprehensive validation of the developed calibration system and its uncertainty model over the full 150 mm range is planned through a future international interlaboratory comparison using suitable transfer standards.

Future work will focus on the validation and implementation of the vision-based alignment system to further improve transducer alignment accuracy and reduce residual alignment errors. In addition, the use of infrared laser interferometry in high-precision displacement metrology is receiving increasing attention [35,36]. The infrared laser interferometer recently acquired in the laboratory will be evaluated as a potential reference standard for the presented calibration system. A particular challenge is the establishment of its metrological traceability, since the interferometer operates at an infrared wavelength of 1550 nm, for which conventional wavelength calibration approaches used for 633 nm He-Ne interferometers cannot be directly applied. Therefore, future work will also address the development of an appropriate traceability procedure for the infrared interferometer. Further improvements in the linear uncertainty component are expected through enhanced control of thermal influences.

## Figures and Tables

**Table 1:** Uncertainty budget.

Quantity *X*_i_	Quantity Estimated Value x_i_	Standard Uncertainty u(x_i_)	Distribution	Sensitivity Coefficient c_i_	Standard Uncertainty Contribution
$L_{T}$	*L*	3 nm	Rectangular	1	3 nm
$\alpha_{T}$	Depending on probe material	$0.58\cdot 10^{-6}\;K^{-1}$	Rectangular	0.1 K·*L*	6·10^−8^·*L*
$\theta_{T}$	0 K	0.02 K	Rectangular	8·10^−6^ K^−1^·*L*	2·10^−7^·*L*
$L_{LI}$	*L*	10 nm + 2·10^−7^·*L*	Normal	1	10 nm + 2·10^−7^·*L*
$e_{cos}$	0	3·10^−7^·*L*	Rectangular	1	3·10^−7^·*L*
$e_{dp}$	0	9 nm	Rectangular	1	9 nm
$e_{cosT}$	0	6·10^−8^·*L*	Rectangular	1	6·10^−8^·*L*
$e_{Abbe}$	0	4 nm	Rectangular	1	4 nm
$e_{re};n=30$	0	2 nm	Normal	1	2 nm
			Total:	$u_{c}=\sqrt{\left(14.5\;\mathrm{nm}{)}^{2}+(4.2\cdot 10^{-7}\cdot L{)}^{2}\right.}$	

## Data Availability

The data presented in this study are not publicly available but can be obtained from the corresponding author upon reasonable request.
